# SIRT3, PP2A and TTP protein stability in the presence of TNF‐α on vincristine‐induced apoptosis of leukaemia cells

**DOI:** 10.1111/jcmm.14949

**Published:** 2020-01-13

**Authors:** Liang‐Jun Wang, Jing‐Ting Chiou, Yuan‐Chin Lee, Chia‐Hui Huang, Yi‐Jun Shi, Long‐Sen Chang

**Affiliations:** ^1^ Institute of Biomedical Sciences National Sun Yat‐Sen University Kaohsiung Taiwan; ^2^ Department of Biotechnology Kaohsiung Medical University Kaohsiung Taiwan

**Keywords:** PP2Acα stabilization, SIRT3 degradation, TNF‐α mRNA decay, TTP degradation, Vincristine

## Abstract

The contribution of vincristine (VCR)‐induced microtubule destabilization to evoke apoptosis in cancer cells remains to be resolved. Thus, we investigated the cytotoxic mechanism of VCR on U937 and HL‐60 human leukaemia cell lines. We discovered that VCR treatment resulted in the up‐regulation of TNF‐α expression and activation of the death receptor pathway, which evoked apoptosis of U937 cells. Moreover, VCR induced microtubule destabilization and mitotic arrest. VCR treatment down‐regulated SIRT3, and such down‐regulation caused mitochondrial ROS to initiate phosphorylation of p38 MAPK. p38 MAPK suppressed MID1‐modulated degradation of the protein phosphatase 2A (PP2A) catalytic subunit. The SIRT3‐ROS‐p38 MAPK‐PP2A axis inhibited tristetraprolin (TTP)‐controlled TNF‐α mRNA degradation, consequently, up‐regulating TNF‐α expression. Restoration of SIRT3 and TTP expression, or inhibition of the ROS‐p38 MAPK axis increased the survival of VCR‐treated cells and repressed TNF‐α up‐regulation. In contrast to suppression of the ROS‐p38 MAPK axis, overexpression of SIRT3 modestly inhibited the effect of VCR on microtubule destabilization and mitotic arrest in U937 cells. Apoptosis of HL‐60 cells, similarly, went through the same pathway. Collectively, our data indicate that the SIRT3‐ROS‐p38 MAPK‐PP2A‐TTP axis modulates TNF‐α expression, which triggers apoptosis of VCR‐treated U937 and HL‐60 cells. We also demonstrate that the apoptotic signalling is not affected by VCR‐elicited microtubule destabilization.

## 1 INTRODUCTION

Microtubule dynamics and stability affect intracellular trafficking, cell division, cellular motility and cell shape maintenance.^1^ The role of microtubules in cell regulation makes them an attractive target for cancer therapy.^2^ Microtubule targeting agents (MTAs) can be divided into microtubule‐destabilizing (*ie* vincristine, vinblastine and nocodazole) and microtubule‐stabilizing agents (*ie* paclitaxel and docetaxel). Particular MTAs cause cell cycle arrest during G2/M phase, activating the apoptotic signalling pathway in tumour cells [2.3]. Additionally, MTAs have been shown to affect cells in interphase (G1).^3^, ^4^, ^5^, ^6^ Thus, the suppression of microtubule dynamics without the accumulation of mitotic cells also induces apoptosis of cancer cells.^2^, ^3^, ^4^, ^5^ Previous studies have suggested that MTAs exert their cytotoxic effects by altering mitochondrial function and cellular signalling, which is independent of the cell cycle.^3^, ^5^, ^6^ Thus, the causal relationship between mitotic arrest and the activation of the apoptotic pathway in MTA‐treated cells remains challenging.

Vincristine (VCR) is a vinca alkaloid from the plant *Catharanthus roseus*, which disrupts microtubule polymerization and induces G2/M cell cycle arrest.^3^ VCR has been used as a chemotherapeutic agent in the treatment of acute lymphoblastic leukaemia (ALL) and certain lymphomas.^3^ The combination of VCR with other chemotherapeutic agents has also been reported to improve the treatment of colorectal cancer, lung cancer and breast cancer.^7^, ^8^, ^9^ Previous studies found that VCR induces G1 arrest and apoptosis in primary ALL cells,^10^ suggesting that VCR could induce apoptosis outside of mitosis. Previous studies also reported that VCR destabilizes the tubulin network in both cervical carcinoma cell lines and breast cancer cell lines; however, VCR only induces apoptosis at the G2/M phase in cervical carcinoma cell lines.^11^ These results highlight the notion that the microtubule destabilization and mitotic arrest effects of VCR are not significantly involved in causing apoptosis. However, the signalling pathway responsible for VCR‐induced apoptosis has yet to be fully understood. Therefore, we investigated the mechanisms through which VCR activates the signalling pathways that induce apoptosis in U937 and HL‐60 human acute myeloid leukaemia (AML) cells.

## 2 MATERIALS AND METHODS

### 2.1 Chemicals and antibodies

Without specific indication, the reagents purchased from Sigma‐Aldrich Inc. were used in the present study, and cell culture supplements were from GIBCO/Life Technologies Inc. Vincristine (VCR), SZLP1‐41 (Skp2 inhibitor), E64D (lysosomal protease inhibitor) and epoxomicin (proteasome inhibitor) were the products of Apexbio Technology LLC. GKT137831 and GLX351322 were purchased from MedChem Express; MitoSOX, annexin V‐FITC/propidium iodide (PI) apoptosis detection kit, tetramethylrhodamine (TMRM) and H_2_DCFDA were from Molecular Probes (Eugene, OR). Antibodies against caspase‐3 and caspase‐8, okadaic acid, Z‐IETD‐FMK and Z‐DEVD‐FMK were purchased from Calbiochem. Antibodies separately against PP2Ac, Mcl‐1, tristetraprolin (TTP), Fas, FasL, TNF‐α and FADD were purchased from Santa Cruz Biotechnology Inc.; and antibody against TNFR1 was from R&D Systems. Antibodies separately against ERK, p‐ERK, p38 MAPK, p‐p38 MAPK, JNK, p‐JNK, SIRT3, TNFR2, α‐tubulin, MID1, α4, caspase‐9, Bax, Bak and Bcl‐2 were obtained from Cell Signaling Technology. Antibodies separately against cytochrome c, Bcl‐xL and Bid were the products of BD Pharmingen Technical (San Jose, CA), and anti‐NOX4 antibody was from Novus Biologicals. Secondary antibodies conjugated with horseradish peroxidase (HRP) were the products of Pierce (Rockford, IL).

### 2.2 Cell culture

Human AML U937 and HL‐60 cells were purchased from BCRC (Hsinchu, Taiwan) and cultured in RPMI‐1640 medium containing 10% FCS (foetal calf serum), 1% sodium pyruvate, 2 mmol/L L‐glutamine, penicillin (100 U/mL) and streptomycin (100 μg/mL). All cell lines were incubated in an incubator humidified with 5% CO_2_ atmosphere. Reduction in the survival of VCR‐treated cells was detected using MTT assay. Apoptotic cell death induced by VCR was detected using annexin V‐FITC/PI kit (Molecular Probes).

### 2.3 Cell cycle analysis

Cell cycle experiments were conducted as previously described.^12^ After treatment with VCR, the cell cycle distribution of treated cells was determined by staining DNA with propidium iodide (PI) followed by flow cytometry.

### 2.4 Detection of ROS generation and mitochondrial membrane potential (ΔΨm)

Vincristine‐treated cells were incubated with 10 μmol/L H_2_DCFDA for 20 min at room temperature. Intracellular ROS levels were measured using Beckman Coulter Paradigm^™^ Detection Platform with excitation at 485 nm and emission at 530 nm. To detect mitochondrial ROS levels, VCR‐treated cells were stained with MitoSOX Red Mitochondrial Superoxide indicator kit according to the manufacturer's protocol (Molecular Probes). Intracellular and mitochondrial ROS levels in VCR‐treated cells were shown as fold increase in fluorescence intensity per microgram of proteins compared with the control group. To measure ΔΨm, VCR‐treated cells were incubated with 2 nmol/L TMRM for 20 min prior to harvesting and then washed with PBS. Fluorescence intensity of TMRM was determined by flow cytometry. Cells with reduced fluorescence (less TMRM) were counted as having lost their ΔΨm.

### 2.5 Preparation of soluble and insoluble tubulin fractions from cells

After treatments with VCR, nocodazole or paclitaxel for 24 h, cells were lysed in 30 μL lysis buffer (20 mmol/L Tris‐HCl, pH 6.8, 1 mmol/L MgCl_2_, 2 mmol/L EGTA, 0.5% NP‐40, 2 mmol/L PMSF and protease inhibitors) for 10 min. The lysates were centrifuged at 13 000 rpm for 10 min at 25°C. The supernatant contained the unpolymerized and soluble tubulin fraction. The pellet was the polymerized tubulin fraction which was resuspended in lysis buffer and sonicated. After centrifugation, the supernatant (insoluble tubulin) was collected. The soluble (S) and insoluble tubulin (P) fractions were subjected to Western blot analyses and detected using anti‐α‐tubulin antibody.

### 2.6 Quantitative RT‐PCR (qRT‐PCR)

Extraction and isolation of total RNA from cells was conducted using the RNeasy mini kit (QIAGEN, Leiden, the Netherlands), and reverse transcription of mRNA was performed using M‐MLV reverse transcriptase (Promega, Madison, WI). Quantitative PCR was performed to detect the levels of TNF‐α, TTP, PP2Acα and SIRT3 mRNA using GoTaq qPCR Master mix (Promega, Madison, WI). The primer sequences used are provided in Table S1.

### 2.7 Stability of TNF‐α mRNA

After specific treatment, cells were incubated with actinomycin D (10 μg/mL) for indicated time periods. The decay of TNF‐α mRNA was measured by qRT‐PCR.

### 2.8 Immunoblotting

Cells were lysed in RIPA buffer containing protease and phosphatase inhibitors. Equal amount of proteins were loaded on SDS‐PAGE and electrophoretically transferred to PVDF membranes. After blocking with 5% non‐fat milk, the PVDF membrane was incubated with primary antibodies, followed by incubation with the appropriate HRP‐labelled secondary antibodies. Enhanced chemiluminescence substrate (Perkin Elmer. Waltham, MA) was used to detect the immunoreactive bands. Western blot analyses were repeated at least three times with similar results.

### 2.9 Analysis of PP2Acα protein stability

After treatment with 5 nmol/L VCR for 24 h, cells were incubated with 10 μmol/L cycloheximide for 1, 2 and 4 h. PP2Acα protein expression was analysed using Western blot analyses.

### 2.10 Transfection of DNA

The plasmids pCMV3‐His‐SIRT3 and pCMV3‐MID1‐HA were the products of Sino Biological Inc (Wayne, PA). The pCMV‐TTP‐HA plasmid was a kind gift from Dr P. Blackshear (National Institute of Environmental Health Sciences, USA). Preparation of pGL‐TNF‐α luciferase promoter construct was described in our previous studies.^13^ Transfection of the plasmids into leukaemia cells was conducted using 4D‐Nucleofector (Lonza Cologne AG, Germany). The dual‐luciferase reporter assay system (Promega, Madison, WI) was used to measure luciferase activity. The luciferase activity was normalized relative to the control *Renilla* luciferase activity.

### 2.11 Knockdown of FADD, α4 and NOX4

FADD siRNA, α4 siRNA, NOX4 siRNA and negative control siRNA were the products of Santa Cruz Biotechnology Inc Transfection of siRNA into cells was performed using Lipofectamine^™^ 2000 according to manufacturer's protocol (Invitrogen).

### 2.12 Measurement of SIRT3 deacetylase activity

SIRT3 deacetylase activity was detected using a SIRT3 Fluorimetric Drug Discovery kit (Enzo Life Sciences Inc, Farmingdale, NY) according to the manufacturer's protocol. In brief, the cell lysate was incubated with the SIRT3 assay buffer and then co‐incubated with Fluoro‐Substrate Peptide, NAD and Developer at 37°C for 1 h. Fluorescent intensity was measured using a fluorescence microplate reader with excitation and emission wavelength at 360 and 460 nm, respectively.

### 2.13 Statistical analysis

All data are presented as mean ± SD. Statistical analyses were conducted using two‐tailed and Student's *t* test, and a *P* < .05 was considered statistically significant. All data presented are results obtained from at least three independent experiments. The β‐actin is used as a loading control, and quantitative analyses of the protein levels are indicated at the immunoblots.

## 3 RESULTS AND DISCUSSION

Concentration‐ and time‐dependent treatment with VCR reduced the survival of U937 cells (Figure S1A). Treatment was completed at a half‐maximal inhibitory concentration (IC_50_) of approximately 5 nmol/L for 24 h. Thus, we utilized these parameters of VCR to investigate VCR's cytotoxic mechanism. Figure S1B shows that VCR induced U937 cell accumulation during the G2/M phase and increased the sub‐G1 cell population. VCR and nocodazole (a microtubule destabilizer) suppressed tubulin polymerization, whereas paclitaxel (a microtubule stabilizer) increased tubulin polymerization (Figure S1C). Such polymerization ostensibly revealed the microtubule‐destabilizing effect of VCR at G2/M arrest. VCR treatment increased the numbers of cells stained with annexin V‐FITC (Figure S1D). VCR‐treated cells showed the cleavage of procaspase‐3/‐8/‐9 (Figure S1E). The caspase inhibitors (Z‐IETD‐FMK and Z‐DEVD‐FMK) inhibited VCR‐induced death of U937 cells (Figure S1F). Thus, VCR has been shown to induce apoptosis in U937 cells.

Numerous studies have highlighted that the association between the loss of the mitochondrial transmembrane potential to apoptosis.^14^ Treatment of U937 cells with VCR depleted the mitochondrial membrane potential (ΔΨm) (Figure S2A) and increased the release of mitochondrial cytochrome c into cytosol (Figure S2B). In the mitochondrial pathway of apoptosis, cleavage of Bid by caspase‐8 produces a truncated Bid (tBid), causing a disruption in the ΔΨm.^15^ VCR treatment increased the production of tBid as well as reduced Bcl‐2 and Bcl‐xL expression in U937 cells (Figure S2C). The death receptor‐mediated pathway is related to FADD‐associated auto‐cleavage and activation of procaspase‐8, which activates caspase‐3 and the cell death pathway.^16^ The knockdown of FADD using siRNA inhibited the cleavage of Bid and the degradation of procaspase‐8/‐3 in VCR‐treated cells (Figure S2D). Additionally, the down‐regulation of FADD increased the survival of VCR‐treated cells (Figure S2E). These results revealed the association of the death receptor‐mediated pathway with VCR‐induced apoptosis in U937 cells.

**Figure 1:**
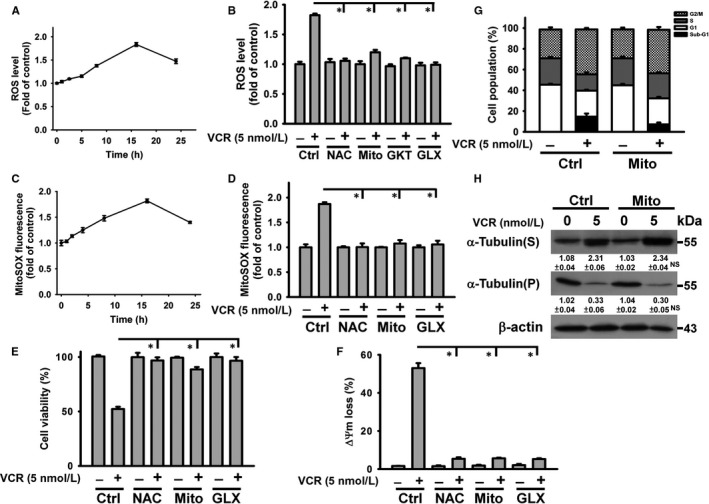
VCR‐induced mitochondrial ROS generation contributed to its cytotoxicity in U937 cells. Without specific indication, U937 cells were treated with 5 nmol/L VCR for 24 h. U937 cells were pre‐treated with 2 mmol/L N‐acetylcysteine (NAC), 50 μmol/L GKT137831 (GKT), 10 μmol/L GLX351322 (GLX) or 10 μmol/L Mito‐TEMPO (Mito) for 1 h and then incubated with 5 nmol/L VCR for 24 h. A, VCR induced an increase in ROS generation. U937 cells were incubated with VCR for indicated time periods. Results were shown as fold increase in fluorescence intensity compared with the control group. Each value is the mean ± SD of three independent experiments with triplicate measurements. ROS was quantified by the fluorescence plate reader. B, Effect of NAC, GKT137831, GLX351322 and Mito‐TEMPO on VCR‐induced ROS generation. U937 cells were treated with 5 nmol/L VCR for 16 h. The data represent the mean ± SD (*P < .05). C, Measurement of mitochondrial ROS generation using mitochondrial superoxide probe MitoSOX Red. U937 cells were incubated with VCR for indicated time periods. The data represent the mean ± SD. D, Effect of NAC, GLX351322 and Mito‐TEMPO on the production of mitochondrial ROS in U937 cells after VCR treatment for 16 h (mean ± SD, *P < .05). E, Effect of NAC, GLX351322 and Mito‐TEMPO on the viability of VCR‐treated cells (mean ± SD, *P < .05). F, Effect of NAC, GLX351322 and Mito‐TEMPO on VCR‐induced ΔΨm loss (mean ± SD, *P < .05). G, Effect of Mito‐TEMPO on cell cycle phase distribution of VCR‐treated cells. H, Effect of Mito‐TEMPO on tubulin polymerization in VCR‐treated cells (NS, statistically insignificant, Mito‐TEMPO/VCR‐treated cells compared to VCR‐treated cells)

Prior studies have reported on the cytotoxicity of VCR and its relation to the induction of ROS generation.^17^ Thus, we measured the ROS levels in VCR‐treated U937 cells. VCR induced a maximal ROS production after 16 h of treatment (Figure 1A). Intracellular ROS is generally produced from mitochondria or NADPH oxidase (NOX).^18^, ^19^ Pre‐treatment with NAC (ROS scavenger), GKT137831 (NOX1/NOX4 inhibitor) or GLX351322 (NOX4 inhibitor) significantly reduced VCR‐induced ROS generation in U937 cells (Figure 1B). Consistently, previous studies have shown that VCR‐induced ROS generation is associated with the activation of NADPH oxidase.^20^ As GLX351322 and GKT137831 similarly inhibited VCR‐induced ROS generation, NOX4 may play a significant role in VCR activity. Figure 1C shows an increase in mitochondria ROS production in VCR‐treated cells as demonstrated by measurement of MitoSOX Red fluorescence. Pre‐treatment with NAC, GLX351322 or Mito‐TEMPO (mitochondria‐targeted antioxidant) repressed mitochondrial ROS generation in VCR‐treated U937 (Figure 1D) and the inhibition of VCR on cell viability (Figure 1E). Moreover, the VCR‐induced loss of ΔΨm was suppressed by NAC, GLX351322 or Mito‐TEMPO (Figure 1F). Previous studies have revealed that NOX4 is localized in the mitochondria of various cell types and is involved in mitochondrial dysregulation.^21^, ^22^ These results indicated that VCR‐targeted NOX4 was located at an upstream position for promoting mitochondrial ROS generation and mitochondrial dysregulation in U937 cells. Thus, VCR‐induced mitochondrial ROS generation was associated with its cytotoxicity. Mito‐TEMPO also markedly inhibited ROS generation in VCR‐treated cells (Figure 1B), suggesting that the majority of the ROS is derived from mitochondria in VCR‐treated cells. Pre‐treatment with Mito‐TEMPO reduced the sub‐G1 cell population in VCR‐treated cells but did not affect VCR‐induced G2/M arrest (Figure 1G). Moreover, the microtubule‐destabilizing activity of VCR was not affected by Mito‐TEMPO (Figure 1H). These results indicated that VCR‐induced microtubule depolymerization was not related to its effect on mitochondrial ROS generation.

**Figure 2:**
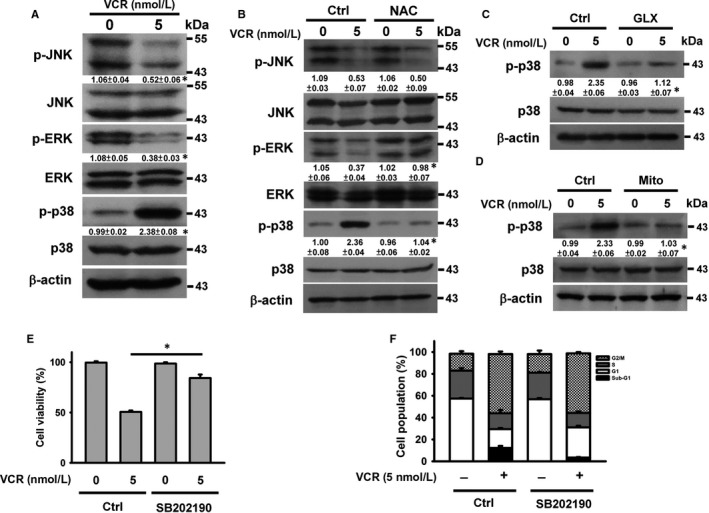
VCR‐induced mitochondrial ROS generation elicited p38 MAPK phosphorylation in U937 cells. Without specific indication, U937 cells were treated with 5 nmol/L VCR for 24 h. U937 cells were pre‐treated with 2 mmol/L N‐acetylcysteine (NAC), 10 μmol/L GLX351322, 10 μmol/L Mito‐TEMPO or 10 μmol/L SB202190 for 1 h and then incubated with 5 nmol/L VCR for 24 h. A, Western blot analyses of phosphorylated MAPKs in VCR‐treated cells (*P < .05, VCR‐treated cells compared to untreated control cells). Effect of NAC (B), GLX351322 (C) and Mito‐TEMPO (D) on the level of phospho‐p38 MAPK in VCR‐treated cells (*P < .05, NAC/VCR‐treated cells compared to VCR‐treated cells; GLX351322/VCR‐treated cells compared to VCR‐treated cells; Mito‐TEMPO/VCR‐treated cells compared to VCR‐treated cells). E, Effect of SB202190 on the viability of VCR‐treated cells (mean ± SD, *P < .05). F, Effect of SB202190 on cell cycle phase distribution of VCR‐treated cells

As VCR‐induced MAPK phosphorylation has been reported to be involved in the degradation of procaspase‐3,^23^ the involvement of MAPK in VCR cytotoxicity was analysed. Treatment with VCR increased the phosphorylation of p38 MAPK and decreased the levels of phosphorylated ERK and JNK (Figure 2A). Pre‐treatment with NAC, GLX351322 and Mito‐TEMPO inhibited p38 MAPK phosphorylation (Figure 2B, 2C and 2D), suggesting that VCR‐induced ROS production induces p38 MAPK activation in U937 cells. NAC pre‐treatment restored p‐ERK levels in VCR‐treated cells, while p‐JNK levels were not affected (Figure 2B). Prior studies have consistently shown that activated p38 MAPK causes dephosphorylation of ERK in U937 cells.^24^ SB202190 pre‐treatment reduced the cell death (Figure 2E) and sub‐G1 population (Figure 2F) induced by VCR, indicating that VCR cytotoxicity is mediated through mitochondrial ROS‐triggered p38 MAPK activation. However, SB202190 did not affect VCR‐induced mitotic arrest (Figure 2F).

**Figure 3:**
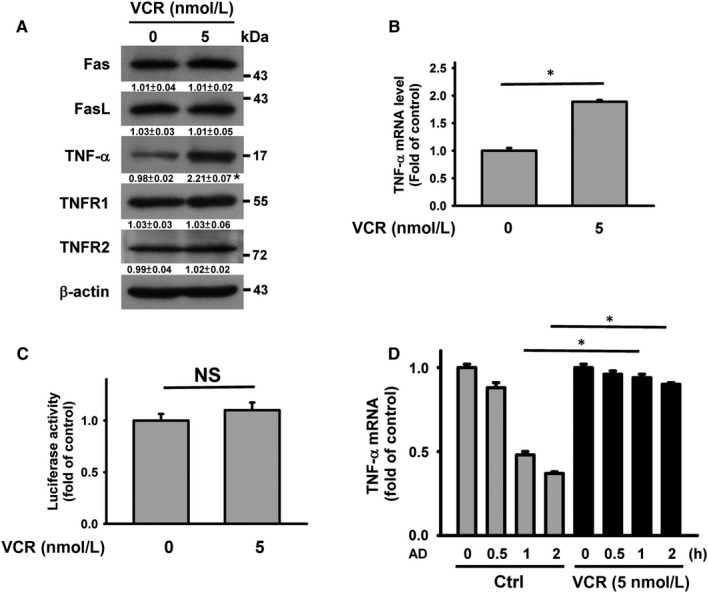
VCR induced up‐regulation of TNF‐α protein expression in U937 cells. Without specific indication, U937 cells were treated with 5 nmol/L VCR for 24 h. A, Effect of VCR on TNF‐α, TNFR1, TNFR2, Fas and FasL protein expression (*P < .05, VCR‐treated cells compared to untreated control cells). B, qRT‐PCR analyses of TNF‐α mRNA level in VCR‐treated cells. The values represent averages of three independent experiments with triplicate measurements (mean ± SD, *P < .05). C, Effect of VCR on the luciferase activity of TNF‐α promoter construct. TNF‐α promoter construct‐transfected cells were treated with VCR for 24 h and then harvested for measuring luciferase activity (NS, statistically insignificant). D, Effect of VCR treatment on TNF‐α mRNA stability. Cells were treated with or without VCR for 24 h and then incubated with 10 μg/mL actinomycin D (AD) for the indicated time periods. The level of TNF‐α mRNA was analysed by qRT‐PCR. VCR‐untreated and VCR‐treated cells without AD treatment were used as control (mean ± SD, *P < .05)

The above results showed that the death receptor pathway modulated apoptosis in VCR‐treated U937 cells. Therefore, we sought to analyse the expression of TNF‐α family proteins. VCR treatment increased TNF‐α protein expression, whereas the expression of TNF‐α receptors, Fas and FasL was unaffected (Figure 3A). Compared to untreated control cells, TNF‐α mRNA expression was elevated in VCR‐treated cells, as revealed by quantitative PCR analyses (Figure 3B). VCR treatment did not considerably affect the pGL‐TNF‐α luciferase activity (Figure 3C) but reduced the degradation of TNF‐α mRNA (Figure 3D). These results revealed that the post‐transcriptional up‐regulation of TNF‐α occurred in VCR‐treated cells.

**Figure 4:**
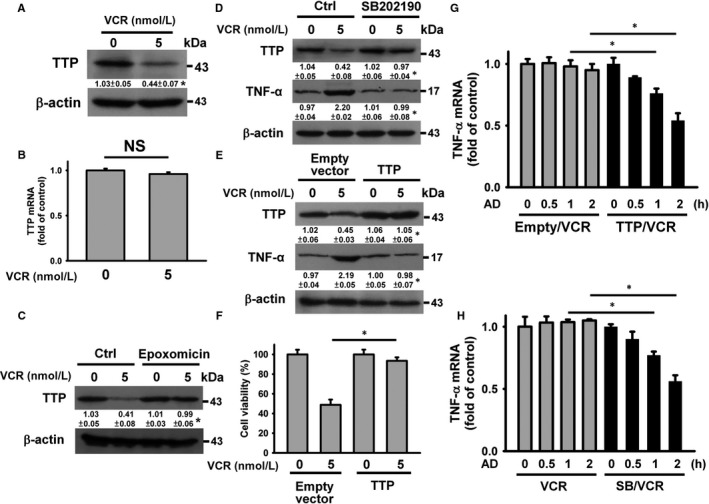
p38 MAPK‐mediated TTP degradation increased TNF‐α mRNA stability in VCR‐treated cells. Without specific indication, U937 cells were treated with 5 nmol/L VCR for 24 h. U937 cells were pre‐treated with 1 μmol/L epoxomicin or 10 μmol/L SB202190 for 1 h and then incubated with 5 nmol/L VCR for 24 h. A, Effect of VCR on TTP protein expression (*P < .05, VCR‐treated cells compared to untreated control cells). B, qRT‐PCR analyses of TTP mRNA level in VCR‐treated cells (NS, statistically insignificant). C, Effect of epoxomicin on VCR‐induced TTP down‐regulation (*P < .05, epoxomicin/VCR‐treated cells compared to VCR‐treated cells). D, Effect of SB202190 on VCR‐induced TTP down‐regulation and TNF‐α up‐regulation (*P < .05, SB202190/VCR‐treated cells compared to VCR‐treated cells). E, Overexpression of TTP inhibited VCR‐induced TNF‐α up‐regulation. After transfection with an empty vector or pCMV‐TTP‐HA for 24 h, the transfected cells were treated with 5 nmol/L VCR for 24 h (*P < .05, VCR‐treated pCMV‐TTP‐HA‐transfected cells compared to VCR‐treated empty vector‐transfected cells). F, Overexpression of TTP restored the viability of VCR‐treated cells. Cell viability was determined using MTT assay (mean ± SD, *P < .05). G, Effect of VCR treatment on TNF‐α mRNA stability in pCMV‐TTP‐HA‐transfected cells. The empty vector‐ and pCMV‐TTP‐HA‐transfected cells were treated with 5 nmol/L VCR for 24 h and then incubated with 10 μg/mL actinomycin D (AD) for the indicated time periods. The level of TNF‐α mRNA was analysed by qRT‐PCR (mean ± SD, *P < .05). The empty vector‐transfected and pCMV‐TTP‐HA‐transfected cells without AD treatment were used as control. H, Effect of SB202190 on TNF‐α mRNA stability in VCR‐treated cells. The level of TNF‐α mRNA was analysed using qRT‐PCR (mean ± SD, *P < .05)

Tristetraprolin (TTP) has been known to promote TNF‐α mRNA turnover.^25^, ^26^ VCR suppressed TTP protein expression in U937 cells (Figure 4A), but TTP mRNA levels remained unchanged (Figure 4B). Proteasome inhibition by epoxomicin mitigated VCR‐induced TTP down‐regulation (Figure 4C), indicating that VCR provokes TTP degradation. In concordance with prior studies investigating the impact of p38 MAPK on TTP degradation,^27^ suppression of p38 MAPK by SB202190 eliminated the effect of VCR on regulating TTP and TNF‐α expression (Figure 4D). Figure 4E shows that TNF‐α up‐regulation did not occur in TTP‐overexpressed cells after VCR treatment. Transfection of U937 cells with pCMV‐TTP‐HA increased the survival of cells exposed to VCR (Figure 4F). In agreement, overexpression of TTP or SB202190 accelerated TNF‐α mRNA decay in U937 cells treated with VCR (Figure 4G and 4H). These observations point to the association between TNF‐α up‐regulation and p38 MAPK‐controlled TTP degradation in VCR‐treated U937 cells.

**Figure 5:**
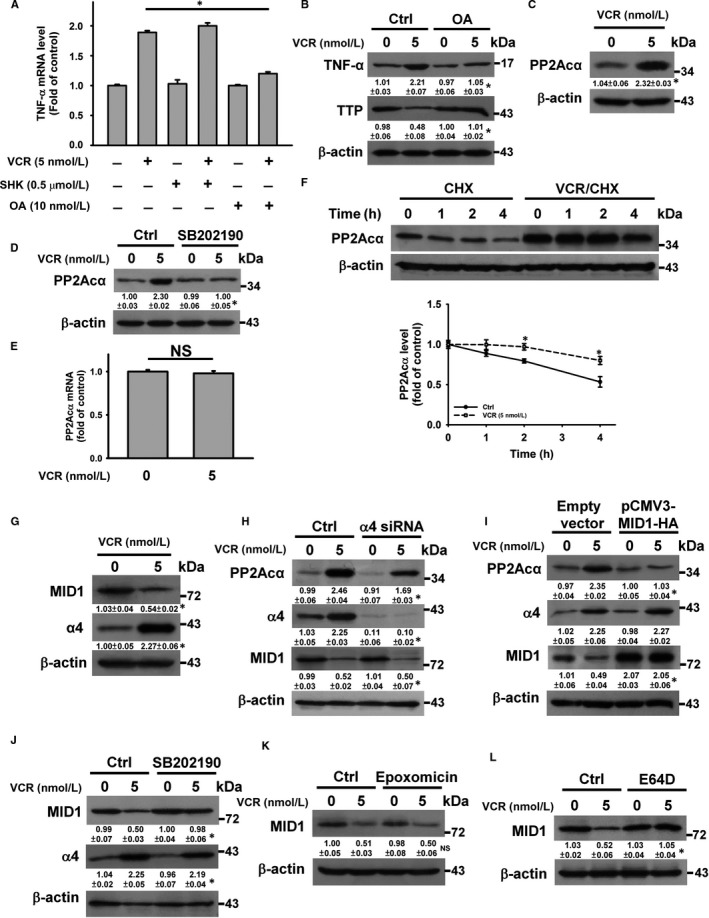
VCR‐induced PP2Acα up‐regulation led to TTP down‐regulation and TNF‐α up‐regulation. Without specific indication, U937 cells were treated with 5 nmol/L VCR for 24 h. U937 cells were pre‐treated with 10 nmol/L okadaic acid (OA), 0.5 μmol/L shikonin (SHK), 10 μmol/L SB202190, 1 μmol/L epoxomicin or 20 μmol/L E64D for 1 h and then incubated with 5 nmol/L VCR for 24 h. A, Effect of okadaic acid and shikonin on the level of TNF‐α mRNA in VCR‐treated cells. B, Effect of OA on TTP and TNF‐α expression in VCR‐treated cells (*P < .05, OA/VCR‐treated cells compared to VCR‐treated cells). C, Effect of VCR on PP2Acα protein expression (*P < .05, VCR‐treated cells compared to untreated control cells). D, Effect of SB202190 on PP2Acα protein expression in VCR‐treated cells (*P < .05, SB202190/VCR‐treated cells compared to VCR‐treated cells). E, qRT‐PCR analyses of PP2Acα mRNA level in VCR‐treated cells (NS, statistically insignificant). F, Turnover of PP2Acα protein in VCR‐treated and VCR‐untreated U937 cells. U937 cells were treated with or without 5 nmol/L VCR for 24 h and then incubated with 10 μmol/L cycloheximide (CHX) for indicated time periods. (Top panel) Western blot analysis. (Bottom panel) Quantification of PP2Acα protein expression from Western blot analyses (*P < .05, compared with control untreated cells). G, Effect of VCR on MID1 and α4 expression (*P < .05, VCR‐treated cells compared to untreated control cells). H, Effect of α4 siRNA on VCR‐induced PP2Acα up‐regulation. U937 cells were transfected with 100 nmol/L control siRNA or α4 siRNA, respectively. After 24 h post‐transfection, the cells were treated with 5 nmol/L VCR for 24 h (*P < .05, VCR‐treated α4 siRNA‐transfected cells compared to VCR‐treated control siRNA‐transfected cells). I, Overexpression of MID1 inhibited VCR‐induced PP2Acα up‐regulation (*P < .05, VCR‐treated pCMV3‐MID1‐HA‐transfected cells compared to VCR‐treated empty vector‐transfected cells). J, Effect of SB202190 on MID1 and α4 expression in VCR‐treated cells (*P < .05, SB202190/VCR‐treated cells compared to VCR‐treated cells). Effect of epoxomicin (K) and E64D (L) on MID1 in VCR‐treated cells (NS, epoxomicin/VCR‐treated cells compared to VCR‐treated cells; *P < .05, E64D/VCR‐treated cells compared to VCR‐treated cells)

Previous studies have demonstrated that the p38 MAPK transcriptionally up‐regulated the expression of protein phosphatase 2A (PP2A) catalytic subunit‐α (PP2Acα), leading to proteasomal degradation of TTP.^27^ Hung et al^28^ suggested that pyruvate kinase M2 (PKM2) plays a role in TTP degradation. In contrast to shikonin (PKM2 inhibitor), okadaic acid (PP2A inhibitor) inhibited the up‐regulation of TNF‐α mRNA expression induced by VCR (Figure 5A). Okadaic acid pre‐treatment mitigated the effect of VCR on TTP protein expression, indicating the role of PP2A in VCR‐induced TTP degradation (Figure 5B). VCR treatment resulted in PP2Acα up‐regulation (Figure 5C), while SB202190 inhibited VCR‐induced up‐regulation of PP2Acα (Figure 5D). VCR did not significantly change PP2Acα mRNA transcription (Figure 5E), while an increase in PP2Acα stability was observed in VCR‐treated cells (Figure 5F). Prior studies suggest that α4 protects PP2A catalytic subunit (PP2Ac) from poly‐ubiquitination and degradation by the microtubule‐associated E3 ubiquitin ligase MID1.^29^, ^30^, ^31^ As shown in Figure 5G, VCR induced α4 up‐regulation and MID1 down‐regulation. The knockdown of α4 partially repressed the VCR‐induced PP2Acα up‐regulation (Figure 5H). Overexpression of MID1 mitigated PP2Acα up‐regulation but not α4 up‐regulation in VCR‐treated cells (Figure 5I). These results confirm that MID1 down‐regulation was mostly responsible for the increased PP2Acα stability in VCR‐treated U937 cells. SB202190 mitigated VCR‐induced MID1 down‐regulation but did not affect VCR‐induced α4 up‐regulation in U937 cells (Figure 5J). These findings suggest that p38 MAPK‐mediated MID1 down‐regulation caused PP2Acα up‐regulation in VCR‐treated U937 cells. Pre‐treatment with epoxomicin did not affect VCR‐induced MID1 down‐regulation (Figure 5K). Previous studies have shown that VCR altered lysosomal integrity and thus induced apoptosis in cancer cells.^11^ Pre‐treatment with chloroquine (lysosome inhibitor) (data not shown) and E64D (lysosomal protease inhibitor) eliminated VCR‐induced MID1 down‐regulation in U937 cells (Figure 5L), indicating that VCR treatment elicited lysosomal degradation of MID1.

**Figure 6:**
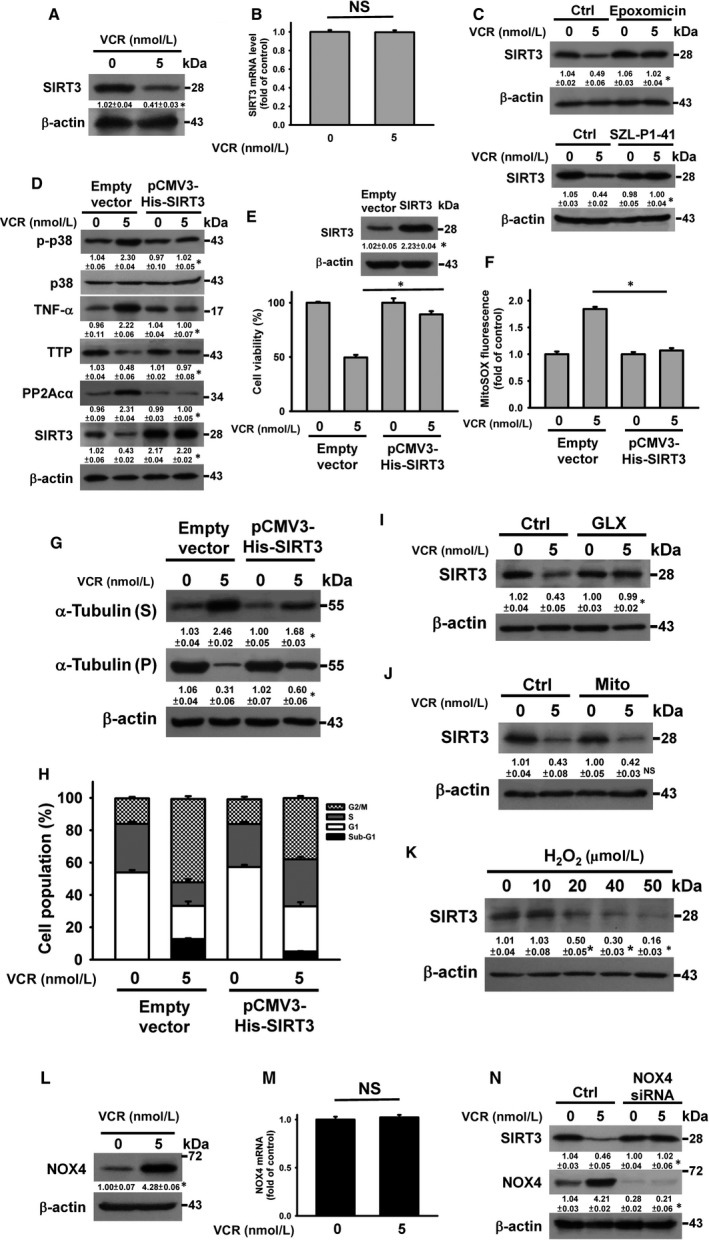
The connection of SIRT3 degradation with VCR‐induced TNF‐α up‐regulation. Without specific indication, U937 cells were treated with 5 nmol/L VCR for 24 h. U937 cells were pre‐treated with 1 μmol/L epoxomicin, 20 μmol/L SZLP1‐41, 10 μmol/L Mito‐TEMPO or 10 μmol/L GLX351322 for 1 h and then incubated with 5 nmol/L VCR for 24 h. A, VCR induced SIRT3 down‐regulation (*P < .05, VCR‐treated cells compared to untreated control cells). B, qRT‐PCR analyses of SIRT3 mRNA level in VCR‐treated cells (NS, statistically insignificant). C, Effect of epoxomicin or SZLP1‐41 on VCR‐induced SIRT3 down‐regulation (*P < .05, epoxomicin/VCR‐treated cells compared to VCR‐treated cells; SZLP1‐41/VCR‐treated cells compared to VCR‐treated cells). D, Effect of SIRT3 overexpression on VCR‐induced p38 MAPK phosphorylation, PP2Acα up‐regulation, TTP down‐regulation and TNF‐α up‐regulation (*P < .05, VCR‐treated pCMV3‐His‐SIRT3‐transfected cells compared to VCR‐treated empty vector‐transfected cells). After transfection with an empty vector or pCMV3‐His‐SIRT3 for 24 h, the transfected cells were treated with 5 nmol/L VCR for 24 h. E. Effect of SIRT3 overexpression on the viability of VCR‐treated cells (mean ± SD, *P < .05). (Inset) SIRT3 expression in empty vector‐ and pCMV3‐His‐SIRT3‐transfected cells (*P < .05, pCMV3‐His‐SIRT3‐transfected cells compared to empty vector‐transfected cells). F, Effect of SIRT3 overexpression on mitochondrial ROS generation in VCR‐treated cells. G, Effect of SIRT3 overexpression on tubulin depolymerization of VCR‐treated cells (*P < .05, VCR‐treated pCMV3‐His‐SIRT3‐transfected cells compared to VCR‐treated empty vector‐transfected cells). H, Effect of SIRT3 overexpression on cell cycle phase distribution of VCR‐treated cells. Effect of GLX351322 (I) and Mito‐TEMPO (J) on SIRT3 expression in VCR‐treated cells (*P < .05, GLX351322/VCR‐treated cells compared to VCR‐treated cells; NS, Mito‐TEMPO/VCR‐treated cells compared to VCR‐treated cells). K, Effect of H_2_O_2_ on SIRT3 expression. U937 cells were treated with indicated H_2_O_2_ concentrations for 24 h (*P < .05, H_2_O_2_‐treated cells compared to untreated control cells). L, Effect of VCR on NOX4 expression in U937 cells (*P < .05, VCR‐treated cells compared to untreated control cells). M, qRT‐PCR analyses of NOX4 mRNA level in VCR‐treated cells (NS, statistically insignificant). N, Effect of NOX4 siRNA on VCR‐induced SIRT3 down‐regulation. U937 cells were transfected with 100 nmol/L control siRNA or NOX4 siRNA, respectively. After 24 h post‐transfection, the cells were treated with 5 nmol/L VCR for 24 h (*P < .05, VCR‐treated NOX4 siRNA‐transfected cells compared to VCR‐treated control siRNA‐transfected cells)

Harkcom et al^32^ found that overexpression of SIRT3 suppresses vinblastine‐induced microtubule destabilization and G_2_/M arrest. Thus, the involvement of SIRT3 in VCR cytotoxicity was analysed. VCR treatment caused down‐regulation of SIRT3 (Figure 6A), while the SIRT3 mRNA levels remained unchanged in VCR‐treated U937 cells (Figure 6B). Pre‐treatment with epoxomicin restored SIRT3 expression in VCR‐treated cells (Figure 6C), indicating that VCR induced SIRT3 degradation. Previous studies have suggested that Skp2 (E3 ubiquitin ligase) regulated SIRT3 degradation.^33^ SZLP1‐41 (Skp2 inhibitor) inhibited VCR‐induced SIRT3 down‐regulation (Figure 6C), suggesting that VCR evoked SIRT3 degradation through the ubiquitin‐proteasome pathway. SIRT3 overexpression revoked the effect of VCR on promoting p38 MAPK‐controlled PP2Acα, TTP and TNF‐α expression (Figure 6D), suggesting that the SIRT3‐p38 MAPK‐PP2A‐TTP axis modulated VCR‐induced TNF‐α up‐regulation. Overexpression of SIRT3 suppressed the cytotoxicity of VCR (Figure 6E). VCR‐induced mitochondrial ROS generation was alleviated by transfection of the pCMV3‐His‐SIRT3 (Figure 6F). It appears that SIRT3 degradation promoted mitochondrial ROS generation when U937 cells were exposed to VCR. These results are in line with previous findings that the deacetylase activity of SIRT3 is involved in SOD2‐mediated scavenging of mitochondrial ROS.^34^, ^35^ Measurement of SIRT3 deacetylase activity showed that VCR treatment reduced the SIRT3 activity (Figure S3A). SIRT3 overexpression reduced microtubule depolymerization (Figure 6G) and mitotic arrest (Figure 6H) in VCR‐treated cells, suggesting that VCR‐induced SIRT3 down‐regulation contributed to the microtubule‐destabilizing effect. GLX351322 (Figure 6I) but not Mito‐TEMPO (Figure 6J) abolished VCR‐induced SIRT3 degradation in VCR‐treated cells, suggesting that NOX4 played a role in VCR‐induced SIRT3 degradation. NOX is known to intracellularly induce the production of H_2_O_2_.^36^ Treatment with H_2_O_2_ caused a reduction in SIRT3 expression (Figure 6K) and SIRT3 deacetylase activity (Figure S3B), suggesting that activated NOX4‐induced H_2_O_2_ production was related to VCR‐induced SIRT3 down‐regulation. Furthermore, the involvement of NOX4 in SIRT3 expression and NOX4 expression was analysed. As shown in Figure 6L, VCR induced NOX4 up‐regulation; however, VCR did not affect NOX4 mRNA levels (Figure 6M). This suggests that VCR post‐translationally up‐regulated NOX4 expression. The knockdown of NOX4 mitigated VCR‐induced SIRT3 down‐regulation (Figure 6N), suggesting a causal role of NOX4 in SIRT3 down‐regulation.

To verify whether the same mechanism was responsible for VCR cytotoxicity on other AML cells, we analysed the death signalling pathway in HL‐60 cells after VCR treatment. VCR suppressed the viability of HL‐60 cells with an IC_50_ value of approximately 1 μmol/L after 24 h (Figure S4A). Treatment of HL‐60 cells with 1 μmol/L VCR induced apoptosis and mitotic arrest (Figure S4B, C). Treatment with VCR notably induced mitochondrial ROS generation and sustained ROS generation was noted after 24 h (Figure S4D). Pre‐treatment with NAC, GLX351322 or Mito‐TEMPO inhibited VCR‐induced mitochondrial ROS generation (Figure S4E). Moreover, GLX351322 and Mito‐TEMPO mitigated VCR‐induced p38 MAPK activation (Figure S4F), indicating that NOX4‐mediated mitochondrial ROS generation was involved in p38 MAPK activation in VCR‐treated HL‐60 cells.

**Figure 7:**
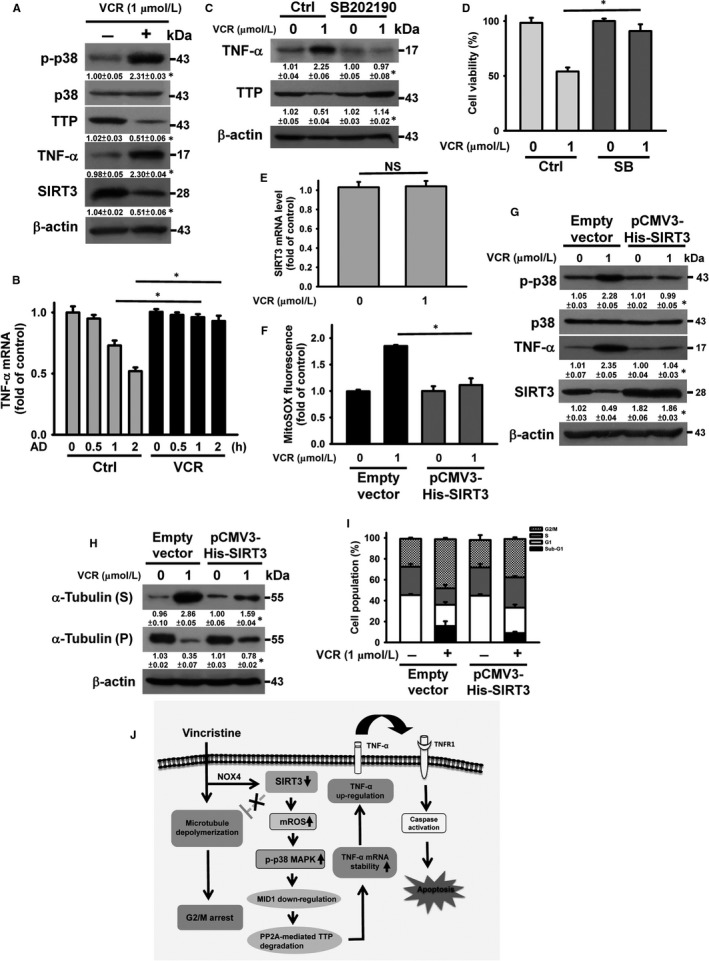
SIRT3/p38 MAPK/TTP axis was involved in VCR‐induced TNF‐α up‐regulation in HL‐60 cells. Without specific indication, HL‐60 cells were treated with 1 μmol/L VCR for 24 h. HL‐60 cells were pre‐treated with 10 μmol/L SB202190 for 1 h and then incubated with 1 μmol/L VCR for 24 h. A, Effect of VCR on the expression of TTP, TNF‐α and SIRT3 in HL‐60 cells (*P < .05, VCR‐treated cells compared to control untreated cells). B, Effect of VCR treatment on TNF‐α mRNA stability. Cells were treated with or without VCR for 24 h and then incubated with 10 μg/ml actinomycin D (AD) for the indicated time periods. The level of TNF‐α mRNA was analysed by qRT‐PCR (mean ± SD, *P < .05). C, Effect of SB202190 on TTP and TNF‐α expression in VCR‐treated cells (*P < .05, SB202190/VCR‐treated cells compared to VCR‐treated cells). D, Effect of SB202190 on the viability of VCR‐treated cells (mean ± SD, *P < .05). E, qRT‐PCR analyses of SIRT3 mRNA level in VCR‐treated cells (NS, statistically insignificant). F, Effect of SIRT3 overexpression on mitochondrial ROS generation in VCR‐treated cells. After transfection with an empty vector or pCMV3‐His‐SIRT3 for 24 h, the transfected cells were treated with 1 μmol/L VCR for 24 h. G, Effect of SIRT3 overexpression on VCR‐induced p38 MAPK phosphorylation and TNF‐α up‐regulation (*P < .05, VCR‐treated pCMV3‐His‐SIRT3‐transfected cells compared to VCR‐treated empty vector‐transfected cells). H, Effect of SIRT3 overexpression on tubulin depolymerization of VCR‐treated cells (*P < .05, VCR‐treated pCMV3‐His‐SIRT3‐transfected cells compared to VCR‐treated empty vector‐transfected cells). I, Effect of SIRT3 overexpression on cell cycle phase distribution of VCR‐treated cells. J, Signalling pathways elucidate activation of TNF‐α‐mediated death pathway in VCR‐treated leukaemia cells. VCR treatment simultaneously induces microtubule depolymerization and NOX4‐mediated SIRT3 degradation. SIRT3 down‐regulation promotes the VCR‐induced microtubule‐destabilizing effect, and microtubule depolymerization evokes G2/M cell cycle arrest in leukaemia cells. Meanwhile, SIRT3 degradation increases the production of mitochondrial ROS, leading to p38 MAPK/MID1/PP2A axis‐mediated TTP degradation in VCR‐treated cells. TTP degradation subsequently attenuates TTP‐mediated TNF‐α mRNA decay. Consequently, TNF‐α up‐regulation and activation of TNF‐α‐mediated death pathway are noted in VCR‐treated cells

Figure 7A, 7B, shows that treatment of HL‐60 cells with VCR increased TNF‐α protein expression and mRNA stability, as well as a reduction in TTP expression. Pre‐treatment with SB202190 eliminated VCR‐induced changes in TNF‐α and TTP expression (Figure 7C) and inhibited cell killing activity of VCR on HL‐60 cells (Figure 7D). These observations indicate that p38 MAPK‐dependent TNF‐α up‐regulation contributed to the VCR‐induced loss of HL‐60 cell survival. VCR treatment induced down‐regulation of SIRT3 protein expression in HL‐60 cells, but SIRT3 mRNA levels were not affected (Figure 7A, 7E). Transfection of pCMV3‐His‐SIRT3 repressed VCR‐induced mitochondrial ROS generation in HL‐60 cells (Figure 7F). VCR was unable to increase p38 MAPK phosphorylation and TNF‐α expression in SIRT3‐overexpressed cells (Figure 7G). SIRT3 overexpression reduced the capability of VCR to induce disassembly of microtubule, G2/M arrest and the accumulation of sub‐G1 cell population (Figure 7H, I). These results indicated that VCR exerted its cytotoxic effect on HL‐60 and U937 cells via the same signalling pathway.

Our data show that VCR elicits NOX4‐mediated SIRT3 degradation in U937 cells (Figure 7J). Previous studies have shown that proteolysis‐inducing factor (PIF) and angiotensin II induce protein degradation through the activation of NADPH oxidase in murine myotube.^37^ Wakatsuki et al 38 reported that activated NADPH oxidase induces the activation of a ubiquitin ligase ZNRF1 (Zinc‐ and RING‐finger 1), which elicits proteasomal degradation of AKT in neurons. Other studies have shown that oxidized proteins are subjected to proteasome degradation.^39^ Thus, it is likely that NOX4‐mediated oxidation of SIRT3 and/or activation of ubiquitin ligase modulates SIRT3 degradation. Consistent with our finding showing H_2_O_2_‐induced SIRT3 down‐regulation in U937 cells, Wang et al 40 found that H_2_O_2_ reduces SIRT3 expression in NSC34 motor neuronal cells. On the contrary, some studies showed that H_2_O_2_ induces SIRT3 up‐regulation in normal melanocyte cell line PIG1,^41^ primary cultured cortical neurons^42^ and HT22 mouse hippocampal cells.^43^ Altogether, these findings suggest that the effect of H_2_O_2_ on SIRT3 expression may be cellular context or cell‐type dependent. Transfection of pCMV3‐His‐SIRT3 reduces the microtubule‐destabilizing effect of VCR in leukaemia cells. Consistent with this, prior studies have revealed that transfection with a SIRT3‐expressed vector reduced the capability of vinblastine to induce disassembly of microtubule polymers in breast cancer MCF‐7 cells.^32^ Notably, Wang et al 12 found that treatment of U937 cells with a SIRT3 inhibitor, brRESV, down‐regulated SIRT3 expression, but did not induce microtubule destabilization and mitotic arrest. These results suggest that SIRT3 down‐regulation might play a role in promoting microtubule destabilization in VCR‐treated cells. On the contrary, the VCR‐induced degradation of SIRT3 leads to mitochondrial ROS generation. Suppression of mitochondrial ROS generation by Mito‐TEMPO did not reduce the VCR‐induced mitotic arrest and microtubule destabilization. These findings confirm that VCR‐induced SIRT3‐mediated mitochondrial ROS generation is distinct from the microtubule‐destabilizing effect of VCR.

Our data further show that the SIRT3‐mitochondrial ROS axis elicits p38 MAPK activation, which increases PP2Acα protein stability; thus, PP2Acα expression in VCR‐treated cells (Figure 7J). Trockenbacher et al 29 reported that the microtubule‐associated E3 ubiquitin ligase MID1 promotes the ubiquitin‐dependent degradation of the PP2Ac. Other studies have demonstrated that α4 is crucially involved in stabilizing PP2Ac and protects PP2Ac from MID1‐mediated degradation.^29^, ^30^, ^31^ VCR treatment induces α4 up‐regulation and lysosomal degradation of MID1 in U937 cells. Compared to α4, MID1 largely contributes to PP2Acα up‐regulation in VCR‐treated U937 cells. Notably, VCR‐induced p38 MAPK activation is involved in MID1 degradation but not α4 up‐regulation (Figure 7J). Unlike previous studies showing that MID1 regulates α4 degradation,^44^ our data show that MID1 overexpression does not affect VCR‐induced α4 up‐regulation. Thus, the mechanism responsible for VCR‐induced α4 up‐regulation needs further investigation.

PP2A is reported to be the phosphatase responsible for the dephosphorylation of TTP,^25^ whereas the dephosphorylated TTP is susceptible to proteasomal degradation.^45^ Accumulating evidence has also shown that TTP functionally accelerates TNF‐α mRNA decay.^25^, ^26^, ^27^, ^28^ In agreement, our data confirmed a causal role of PP2Acα up‐regulation in VCR‐induced TTP down‐regulation. In line with our analysis of the effect of VCR on TTP degradation, up‐regulation of TNF‐α mRNA and protein levels are observed in VCR‐treated U937 and HL‐60 cell lines. Collectively, our data demonstrate that the signalling cascade, SIRT3‐mitochondrial ROS‐p38 MAPK‐TTP axis, modulates TNF‐α expression in VCR‐treated U937 and HL‐60 cell lines (Figure 7J). Notably, previous results have shown that the plasma level of VCR ranges from 90 to 930 nmol/L after the administration of VCR sulphate liposome injection in patients.^46^ Accordingly, the VCR concentration used in the present study is physiologically attainable.

In conclusion, our data show that VCR‐induced TNF‐α up‐regulation causes apoptosis in U937 and HL‐60 cells. Additionally, we have shown that the apoptotic signalling pathway is not related to the microtubule destabilization and mitotic arrest effects of VCR. Regarding previous studies, we are also in agreement that MTA‐induced apoptosis in cancer cells is not exclusively dependent on its effect on mitotic arrest, which is mediated by changes in microtubule organization.^6^, ^17^, ^47^, ^48^ Considering that defects in apoptotic machinery, resulting in the treatment failure of leukaemia,^49^, ^50^ TNF‐α up‐regulation induced by VCR might improve response to apoptotic stimuli in leukaemia. Furthermore, the death pathway activated by VCR provides a novel approach for its optimal utility in combinatorial chemotherapy for leukaemia.

## CONFLICT OF INTERESTS

The authors confirm that there are no conflicts of interest.

## AUTHOR'S CONTRIBUTION

Wang, LJ, Chiou, JT, Lee, YC, Huang, CH and Shi, YJ performed the experiments; Wang, LJ and Chang, LS analysed the data; Wang, LJ and Chang, LS designed the experiments and wrote the paper.

## Data Availability

The data that support the findings of this study are available from the corresponding author upon reasonable request.

## References

[1] Steinmetz MO , Prota AE . Microtubule‐targeting agents: strategies to hijack the cytoskeleton. Trends Cell Biol. 2018;28:776‐792. 29871823 10.1016/j.tcb.2018.05.001

[2] Dumontet C , Jordan MA . Microtubule‐binding agents: a dynamic field of cancer therapeutics. Nat Rev Drug Discov. 2010;9:790‐803. 20885410 10.1038/nrd3253 PMC3194401

[3] Bates D , Eastman A . Microtubule destabilising agents: far more than just antimitotic anticancer drugs. Br J Clin Pharmacol. 2017;83:255‐268. 27620987 10.1111/bcp.13126 PMC5237681

[4] Rovini A , Savry A , Braguer D , Carré M . Microtubule‐targeted agents: when mitochondria become essential to chemotherapy. Biochim Biophys Acta. 2011;1807:679‐688. 21216222 10.1016/j.bbabio.2011.01.001

[5] Mukhtar E , Adhami VM , Mukhtar H . Targeting microtubules by natural agents for cancer therapy. Mol Cancer Ther. 2014;13:275‐284. 24435445 10.1158/1535-7163.MCT-13-0791 PMC3946048

[6] Komlodi‐Pasztor E , Sackett D , Wilkerson J , Fojo T . Mitosis is not a key target of microtubule agents in patient tumors. Nat Rev Clin Oncol. 2011;8:244‐250. 21283127 10.1038/nrclinonc.2010.228

[7] Rainey JM , Jones SE , Salmon SE . Combination chemotherapy for advanced breast cancer utilizing vincristine, adriamycin, and cyclophosphamide (VAC). Cancer. 1979;43:66‐71. 761176 10.1002/1097-0142(197901)43:1<66::aid-cncr2820430109>3.0.co;2-1

[8] Engstrom PF , MacIntyre JM , Douglass HO Jr , et al. Combination chemotherapy of advanced colorectal cancer utilizing 5‐fluorouracil, semustine, dacarbazine, vincristine, and hydroxyurea: a phase III trial by the eastern cooperative oncology group (EST: 4275). Cancer. 1982;49:1555‐1560. 7039814 10.1002/1097-0142(19820415)49:8<1555::aid-cncr2820490807>3.0.co;2-c

[9] Jassem J , Karnicka‐Młodkowska H , Drozd‐Lula M , et al. Combination chemotherapy with vincristine, epirubicin and cyclophosphamide in small cell lung carcinoma. Eur J Cancer. 1992;28:473‐476. 1317199 10.1016/s0959-8049(05)80079-6

[10] Kothari A , Hittelman WN , Chambers TC . Cell cycle‐dependent mechanisms underlie vincristine‐induced death of primary acute lymphoblastic leukemia cells. Cancer Res. 2016;76:3553‐3561. 27197148 10.1158/0008-5472.CAN-15-2104 PMC4911277

[11] Groth‐Pedersen L , Ostenfeld MS , Høyer‐Hansen M , et al. Vincristine induces dramatic lysosomal changes and sensitizes cancer cells to lysosome‐destabilizing siramesine. Cancer Res. 2007;67:2217‐2225. 17332352 10.1158/0008-5472.CAN-06-3520

[12] Wang LJ , Lee YC , Huang CH , et al. Non‐mitotic effect of albendazole triggers apoptosis of human leukemia cells via SIRT3/ROS/p38 MAPK/TTP axis‐mediated TNF‐α upregulation. Biochem Pharmacol. 2019;162:154‐168. 30414389 10.1016/j.bcp.2018.11.003

[13] Chen YJ , Chang LS . Arecoline‐induced death of human leukemia K562 cells is associated with surface up‐modulation of TNFR2. J Cell Physiol. 2012;227:2240‐2251. 21809341 10.1002/jcp.22963

[14] Green DR . Mitochondria and apoptosis. Science. 1998;281:1309‐1312. 9721092 10.1126/science.281.5381.1309

[15] Hengartner MO . The biochemistry of apoptosis. Nature. 2000;407:700‐706. 10.1038/35037710 11048727

[16] Guicciardi ME , Gores GJ . Life and death by death receptors. FASEB J. 2009;23:1625‐1637. 19141537 10.1096/fj.08-111005 PMC2698650

[17] Groninger E , Meeuwsen‐De Boer GJ , et al. Vincristine induced apoptosis in acute lymphoblastic leukaemia cells: a mitochondrial controlled pathway regulated by reactive oxygen species? Int J Oncol. 2002;21:1339‐1345. 12429986 10.3892/ijo.21.6.1339

[18] D'Autréaux B , Toledano MB . ROS as signalling molecules: mechanisms that generate specificity in ROS homeostasis. Nat Rev Mol Cell Biol. 2007;8:813‐824. 17848967 10.1038/nrm2256

[19] Dikalov S . Cross talk between mitochondria and NADPH oxidases. Free Radic Biol Med. 2011;51:1289‐1301. 21777669 10.1016/j.freeradbiomed.2011.06.033 PMC3163726

[20] Alexandre J , Hu Y , Lu W , et al. Novel action of paclitaxel against cancer cells: bystander effect mediated by reactive oxygen species. Cancer Res. 2007;67:3512‐3517. 17440056 10.1158/0008-5472.CAN-06-3914

[21] Kuroda J , Ago T , Matsushima S , et al. NADPH oxidase 4 (Nox4) is a major source of oxidative stress in the failing heart. Proc Natl Acad Sci USA. 2010;107:15565‐15570. 20713697 10.1073/pnas.1002178107 PMC2932625

[22] Bernard K , Logsdon NJ , Miguel V , et al. NADPH oxidase 4 (nox4) suppresses mitochondrial biogenesis and bioenergetics in lung fibroblasts via a nuclear factor erythroid‐derived 2‐like 2 (Nrf2)‐dependent pathway. J Biol Chem. 2017;292:3029‐3038. 28049732 10.1074/jbc.M116.752261 PMC5314196

[23] Stone AA , Chambers TC . Microtubule inhibitors elicit differential effects on MAP kinase (JNK, ERK, and p38) signaling pathways in human KB‐3 carcinoma cells. Exp Cell Res. 2000;254:110‐119. 10623471 10.1006/excr.1999.4731

[24] Liu WH , Chang LS . Caffeine induces matrix metalloproteinase‐2 (MMP‐2) and MMP‐9 down‐regulation in human leukemia U937 cells via Ca^2+^/ROS‐mediated suppression of ERK/c‐fos pathway and activation of p38 MAPK/c‐jun pathway. J Cell Physiol. 2010;224:775‐785. 20432471 10.1002/jcp.22180

[25] Sun L , Stoecklin G , Van Way S , et al. Tristetraprolin (TTP)‐14‐3‐3 complex formation protects TTP from dephosphorylation by protein phosphatase 2a and stabilizes tumor necrosis factor‐α mRNA. J Biol Chem. 2007;282:3766‐3777. 17170118 10.1074/jbc.M607347200

[26] Sugiura R , Satoh R , Ishiwata S , Umeda N , Kita A . Role of RNA‐binding proteins in MAPK signal transduction pathway. J Signal Transduct. 2011;2011:109746. 21776382 10.1155/2011/109746 PMC3135068

[27] Liu WH , Chou WM , Chang LS . p38 MAPK/PP2Acα/TTP pathway on the connection of TNF‐α and caspases activation on hydroquinone‐induced apoptosis. Carcinogenesis. 2013;34:818‐827. 23288922 10.1093/carcin/bgs409

[28] Huang L , Yu Z , Zhang Z , Ma W , Song S , Huang G . Interaction with pyruvate kinase M2 destabilizes tristetraprolin by proteasome degradation and regulates cell proliferation in breast cancer. Sci Rep. 2016;6:22449. 26926077 10.1038/srep22449 PMC4772106

[29] Trockenbacher A , Suckow V , Foerster J , et al. MID1, mutated in Opitz syndrome, encodes an ubiquitin ligase that targets phosphatase 2A for degradation. Nat Genet. 2001;29:287‐294. 11685209 10.1038/ng762

[30] Kong M , Ditsworth D , Lindsten T , Thompson CB . α4 is an essential regulator of PP2A phosphatase activity. Mol Cell. 2009;36:51‐60. 19818709 10.1016/j.molcel.2009.09.025 PMC2761955

[31] Jiang L , Stanevich V , Satyshur KA , et al. Structural basis of protein phosphatase 2A stable latency. Nat Commun. 2013;4:1699. 23591866 10.1038/ncomms2663 PMC3644067

[32] Harkcom WT , Ghosh AK , Sung MS , et al. NAD^+^ and SIRT3 control microtubule dynamics and reduce susceptibility to antimicrotubule agents. Proc Natl Acad Sci USA. 2014;111:E2443‐E2452. 24889606 10.1073/pnas.1404269111 PMC4066477

[33] Iwahara T , Bonasio R , Narendra V , Reinberg D . SIRT3 functions in the nucleus in the control of stress‐related gene expression. Mol Cell Biol. 2012;32:5022‐5034. 23045395 10.1128/MCB.00822-12 PMC3510539

[34] Chen Y , Zhang J , Lin Y , et al. Tumour suppressor SIRT3 deacetylates and activates manganese superoxide dismutase to scavenge ROS. EMBO Rep. 2011;12:534‐541. 21566644 10.1038/embor.2011.65 PMC3128277

[35] Ansari A , Rahman MS , Saha SK , et al. Function of the SIRT3 mitochondrial deacetylase in cellular physiology, cancer, and neurodegenerative disease. Aging Cell. 2017;16:4‐16. 27686535 10.1111/acel.12538 PMC5242307

[36] Nauseef WM . Detection of superoxide anion and hydrogen peroxide production by cellular NADPH oxidases. Biochim Biophys Acta. 2014;1840:757‐767. 23660153 10.1016/j.bbagen.2013.04.040 PMC3770773

[37] Russell ST , Eley H , Tisdale MJ . Role of reactive oxygen species in protein degradation in murine myotubes induced by proteolysis‐inducing factor and angiotensin II. Cell Signal. 2007;19:1797‐1806. 17532611 10.1016/j.cellsig.2007.04.003

[38] Wakatsuki S , Araki T . NADPH oxidases promote apoptosis by activating ZNRF1 ubiquitin ligase in neurons treated with an exogenously applied oxidant. Commun Integr Biol. 2016;9:e1143575. 27195063 10.1080/19420889.2016.1143575 PMC4857788

[39] Pajares M , Jiménez‐Moreno N , Dias IHK , et al. Redox control of protein degradation. Redox Biol. 2015;6:409‐420. 26381917 10.1016/j.redox.2015.07.003 PMC4576413

[40] Wang J , Feng H , Zhang J , et al. Lithium and valproate acid protect NSC34 cells from H_2_O_2_‐induced oxidative stress and upregulate expressions of SIRT3 and CARM1. Neuro Endocrinol Lett. 2013;34:648‐654. 24464007

[41] Yi X , Guo W , Shi Q , et al. SIRT3‐dependent mitochondrial dynamics remodeling contributes to oxidative stress‐induced melanocyte degeneration in vitiligo. Theranostics. 2019;9:1614‐1633. 31037127 10.7150/thno.30398 PMC6485185

[42] Dai SH , Chen T , Wang YH , et al. Sirt3 protects cortical neurons against oxidative stress via regulating mitochondrial Ca^2+^ and mitochondrial biogenesis. Int J Mol Sci. 2014;15:14591‐14609. 25196599 10.3390/ijms150814591 PMC4159870

[43] Dai SH , Chen T , Wang YH , et al. Sirt3 attenuates hydrogen peroxide‐induced oxidative stress through the preservation of mitochondrial function in HT22 cells. Int J Mol Med. 2014;34:1159‐1168. 25090966 10.3892/ijmm.2014.1876

[44] Du H , Huang Y , Zaghlula M , et al. The MID1 E3 ligase catalyzes the polyubiquitination of alpha4 (α4), a regulatory subunit of protein phosphatase 2A (PP2A): novel insights into MID1‐mediated regulation of PP2A. J Biol Chem. 2013;288:21341‐21350. 23740247 10.1074/jbc.M113.481093 PMC3774402

[45] Brook M , Tchen CR , Santalucia T , et al. Posttranslational regulation of tristetraprolin subcellular localization and protein stability by p38 mitogen‐activated protein kinase and extracellular signal‐regulated kinase pathways. Mol Cell Biol. 2006;26:2408‐2418. 16508015 10.1128/MCB.26.6.2408-2418.2006 PMC1430283

[46] Yang F , Jiang M , Lu M , et al. Pharmacokinetic behavior of vincristine and safety following intravenous administration of vincristine sulfate liposome injection in chinese patients with malignant lymphoma. Front. Pharmacol. 2018;9:991. 30210349 10.3389/fphar.2018.00991 PMC6123375

[47] Fürst R , Vollmar AM . A new perspective on old drugs: non‐mitotic actions of tubulin‐binding drugs play a major role in cancer treatment. Pharmazie. 2013;68:478‐483. 23923626

[48] Risinger AL , Dybdal‐Hargreaves NF , Mooberry SL . Breast cancer cell lines exhibit differential sensitivities to microtubule‐targeting drugs independent of doubling time. Anticancer Res. 2015;35:5845‐5850. 26504006 PMC4812601

[49] Rumjanek VM , Vidal RS , Maia RC . Multidrug resistance in chronic myeloid leukaemia: how much can we learn from MDR‐CML cell lines? Biosci Rep. 2013;33:e00081. 24070327 10.1042/BSR20130067 PMC3839595

[50] Bhola PD , Mar BG , Lindsley RC , et al. Functionally identifiable apoptosis‐insensitive subpopulations determine chemoresistance in acute myeloid leukemia. J Clin Invest. 2016;126:3827‐3836. 27599292 10.1172/JCI82908 PMC5096802

