# Supplementary material for: SIRT3, PP2A and TTP protein stability in the presence of TNF‐α on vincristine‐induced apoptosis of leukaemia cells
Source: J Cell Mol Med. 2020 Jan 13;24(4):2552–65. doi: 10.1111/jcmm.14949 (PMC7028858; doi:10.1111/jcmm.14949)

Supplementary Table S1. Primers used for qRT-PCR.

| TNF-α (forward) | 5'-CTGGAGAAGGGTGACCGACTCAG-3' |
| --- | --- |
| TNF-α (reverse) | 5'-TAGA CCTGCCCAGACTCGGCAAAG-3' |
| TTP (forward) | 5′-CATCCACAACCCTA GCGAAGACCTG-3′ |
| TTP (reverse) | 5′-CAGAGAAGGCAGAGGGTGACAGTG-3′ |
| PP2Acα (forward) | 5′-TCGTTGTGGTAACCAAGCTG-3′ |
| PP2Acα (reverse) | 5′-AACATGTGGCTCGCCTCTAC-3′ |
| SIRT3 (forward) | 5′-GACATTCGGGCTGACGTGATGGC-3′ |
| SIRT3 (reverse) | 5′-CAACCACATGCAGCAAGAACCTCTG-3′ |
| NOX4 (forward) | 5′-GGCTGGAGGCATTGGAGTAA-3′ |
| NOX4 (reverse) | 5′-CCAGTCATCCAACAGGGTGTT-3′ |
| GAPDH (forward) | 5'-GAAATCCCATCACCATCTTCCAGG-3' |
| GAPDH (reverse) | 5'-GAGCCCCAGCCTTCTCCATG-3' |

.

Supplementary figure legends

Fig. S1. Vincristine (VCR) induces apoptotic death of U937 cells.

Without specific indication, U937 cells were incubated with 5 nM VCR for 24 h. (A) Concentration-dependent and time-dependent effect of VCR on cell viability. (Inset) Time-dependent effect of VCR on cell viability. U937 cells were incubated with 5 nM VCR for indicated time periods. Cell viability was determined using MTT assay. Results are expressed as the percentage of cell proliferation relative to the control. Each value is the mean±SD of three independent experiments with triplicate measurements. (B) Cell cycle analysis of VCR-treated U937 cells. Flow cytometry analyses showed an increase in the sub-G1 DNA content and G2/M cell cycle arrest of U937 cells (1 × 10^4^ cells) after treatment with 5 nM VCR for 24 h. (C) Effect of VCR, nocodazole, and paclitaxel on tubulin polymerization. U937 cells were treated with 0.25 μM nocodazole (Noc), 5 nM VCR or 0.2 μM paclitaxel (PTX) for 24 h, respectively. Then, the cells were lysed and fractionated from cytosol (supernatant, S) to cytoskeletal (pellet, P) extracts. The extracts were subjected to western blot analysis for α-tubulin and β-actin analysis. Results of western blots were quantified by a scanning densitometer. Changes in protein levels relative to the β-actin loading control are shown at the bottom of immunoreactive bands (**P*<0.05, MTA-treated cells compared to untreated control cells). (D) Flow cytometry analyses of annexin V-PI double staining VCR-treated cells (1 × 10^4^ cells). On the flow cytometric scatter graphs, the left lower quadrant represents remaining live cells. The right lower quadrant represents the population of early apoptotic cells. The right upper quadrant represents the accumulation of late apoptotic cells. (E) Western blot analyses of degradation of procaspase-3/-8/-9 in VCR-treated cells (**P*<0.05, VCR-treated cells compared to untreated control cells). (F) Viability of VCR-treated cells was rescued by pretreatment with caspase inhibitors. U937 cells were pretreated with 10 μM Z-IETD-FMK (caspase-8 inhibitor) or Z-DEVD-FMK (caspase-3 inhibitor) for 1 h, and then incubated with VCR for 24 h. Each value is the mean±SD of three independent experiments with triplicate measurements (**P*<0.05).

Fig. S2. Effect of VCR on mitochondrial membrane potential and Bcl-2 family protein expression

Without specific indication, U937 cells were incubated with 5 nM VCR for 24 h. (A) VCR induced loss of mitochondrial membrane potential (ΔΨm). ΔΨm of VCR-treated cells (1 × 10^4^) was analyzed by flow cytometry. (B) Western blot analyses showing the release of cytochrome c into cytosol in VCR-treated cells (**P*<0.05, VCR-treated cells compared to untreated control cells). (C) Western blot analyses showing the production of t-Bid and expression of Bcl-2 family proteins in VCR-treated cells (**P*<0.05, VCR-treated cells compared to untreated control cells). (D) FADD siRNA abrogated VCR-induced degradation of procaspase-8 and the production of t-Bid. U937 cells were transfected with 100 nM control siRNA or FADD siRNA, respectively. After 24 h post-transfection, the cells were treated with 5 nM VCR for 24 h (**P*<0.05, VCR-treated FADD siRNA-transfected cells compared to VCR-treated control siRNA-transfected cells). (E) Viability of control siRNA- and FADD siRNA-transfected cells after treatment with VCR for 24 h. Cell viability was determined using MTT (mean±SD, **P*<0.05).

Fig. S3. Effect of VCR and H_2_O_2_ on SIRT3 deacetylase activity in U937 cells.

U937 cells were treated with 5 nM VCR (A) or 20 μM H_2_O_2_ (B) for 24 h. SIRT3 deacetylase activity was detected using a SIRT3 Fluorimetric Drug Discovery kit (Enzo Life Sciences Inc., Farmingdale, NY). A control experiment was also conducted using 5 mM suramin (a SIRT inhibitor) provided in the kit. Data represent mean± SD (**P*<0.05).

Fig. S4. VCR-induced mitochondrial ROS generation elicited apoptosis of HL-60 cells.

Without specific indication, HL-60 cells were treated with 1 μM VCR for 24 h. HL-60 cells were pre-treated with 2 mM N-acetylcysteine (NAC), 10 μM GLX351322 (GLX), or 10 μM Mito-TEMPO (Mito) for 1 h, and then incubated with 1 μM VCR for 24 h. (A) Concentration-dependent effect of VCR on cell viability. Cell viability was determined using MTT assay. (B) Flow cytometry analyses of annexin V-PI double staining VCR-treated cells (1 × 10^4^ cells). (C) Cell cycle analysis of VCR-treated HL-60 cells (1 × 10^4^ cells). Flow cytometry analyses showed an increase in the sub-G1 DNA content and G2/M cell cycle arrest of HL-60 cells after treatment with 1 μM VCR for 24 h. (D) Measurement of mitochondrial ROS generation using mitochondrial superoxide probe MitoSOX Red. HL-60 cells were incubated with VCR for indicated time periods. The data represent the mean±SD. (E) Effect of NAC, GLX351322, and Mito-TEMPO on the production of mitochondrial ROS in VCR-treated cells (mean±SD, **P*<0.05). (F) Effect of Mito-TEMPO and GLX351322 on VCR-induced p38 MAPK phosphorylation (**P*<0.05, Mito-TEMPO/VCR-treated cells compared to VCR-treated cells; GLX351322/VCR-treated cells compared to VCR-treated cells).


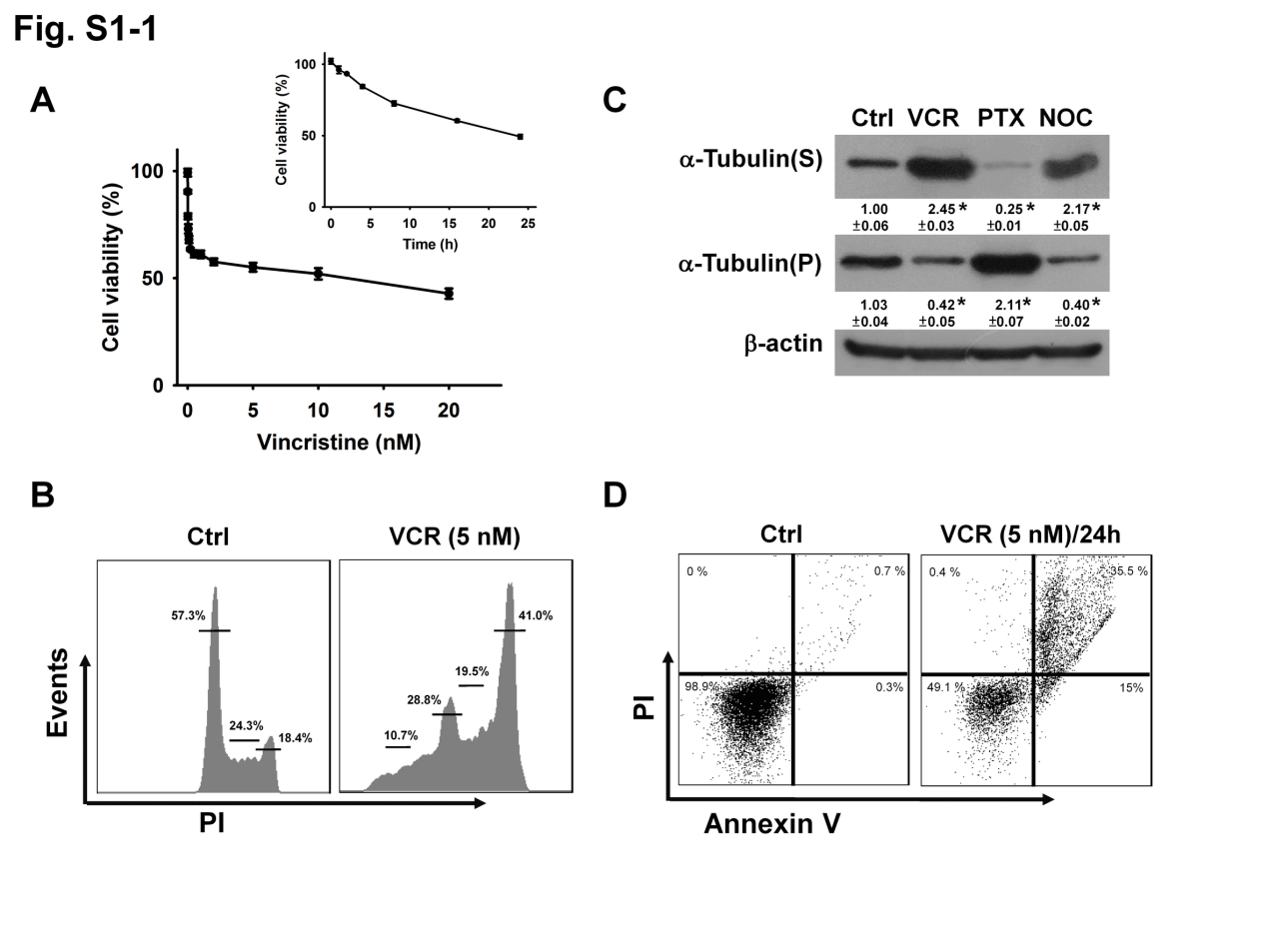


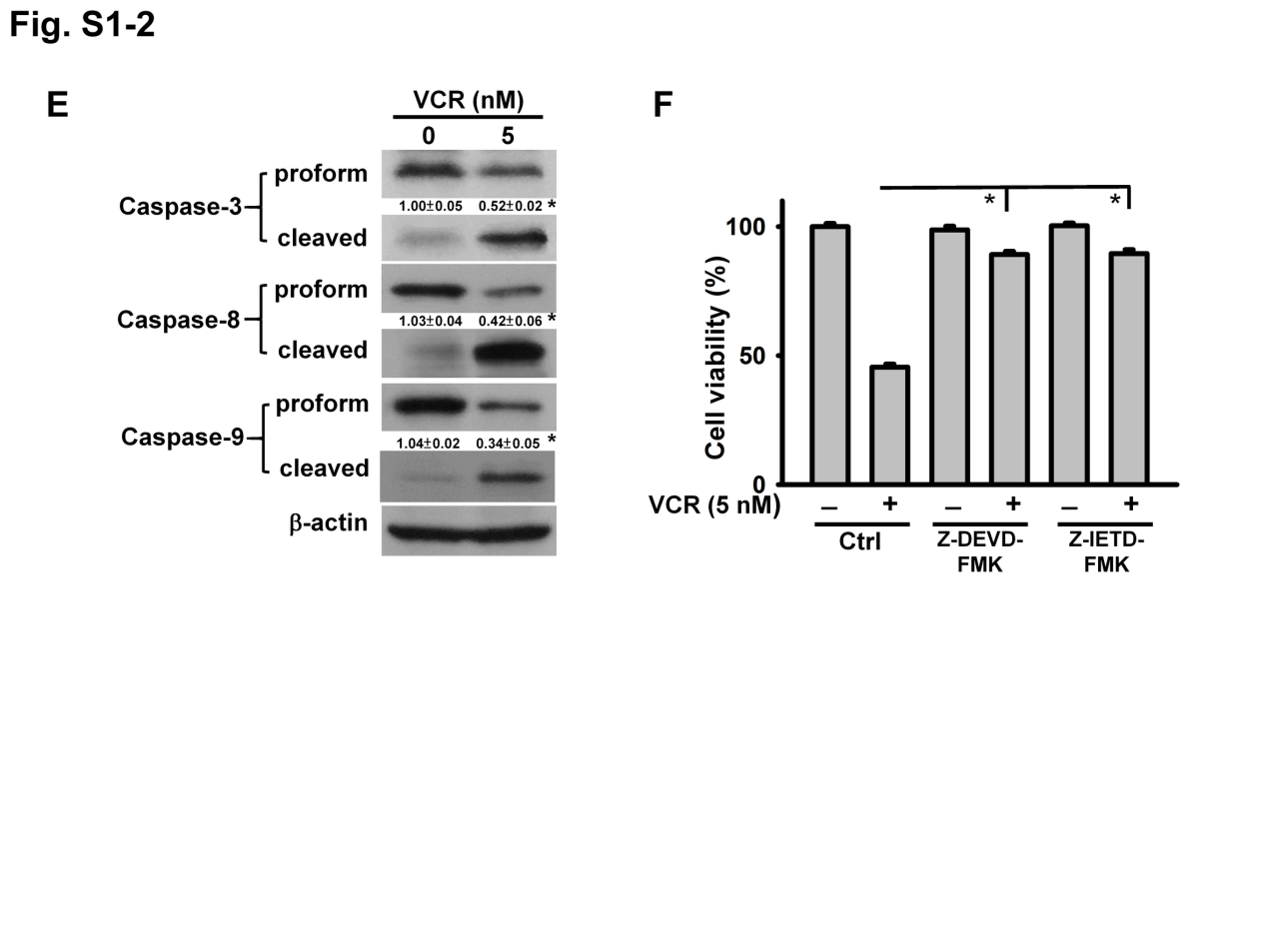


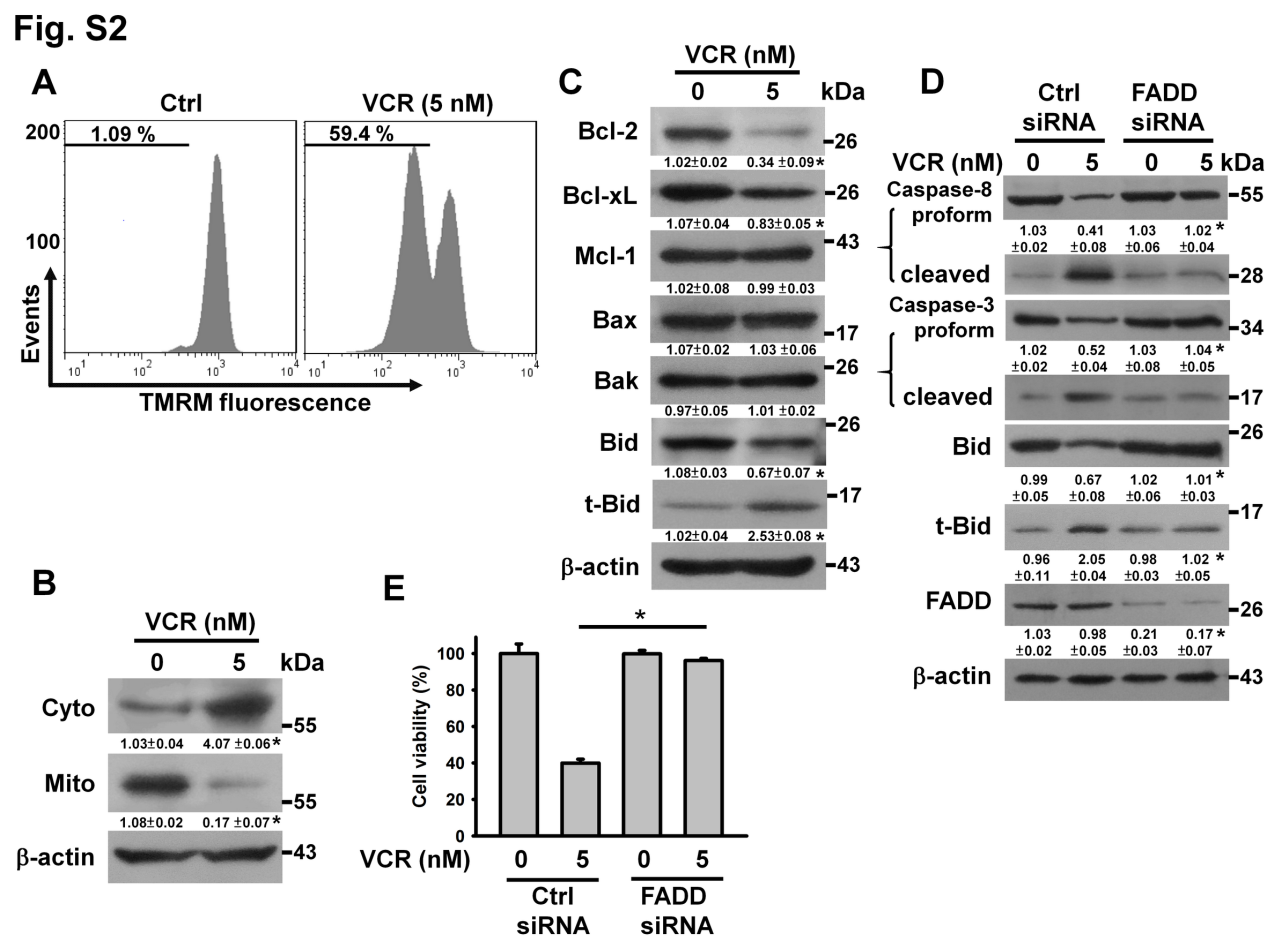


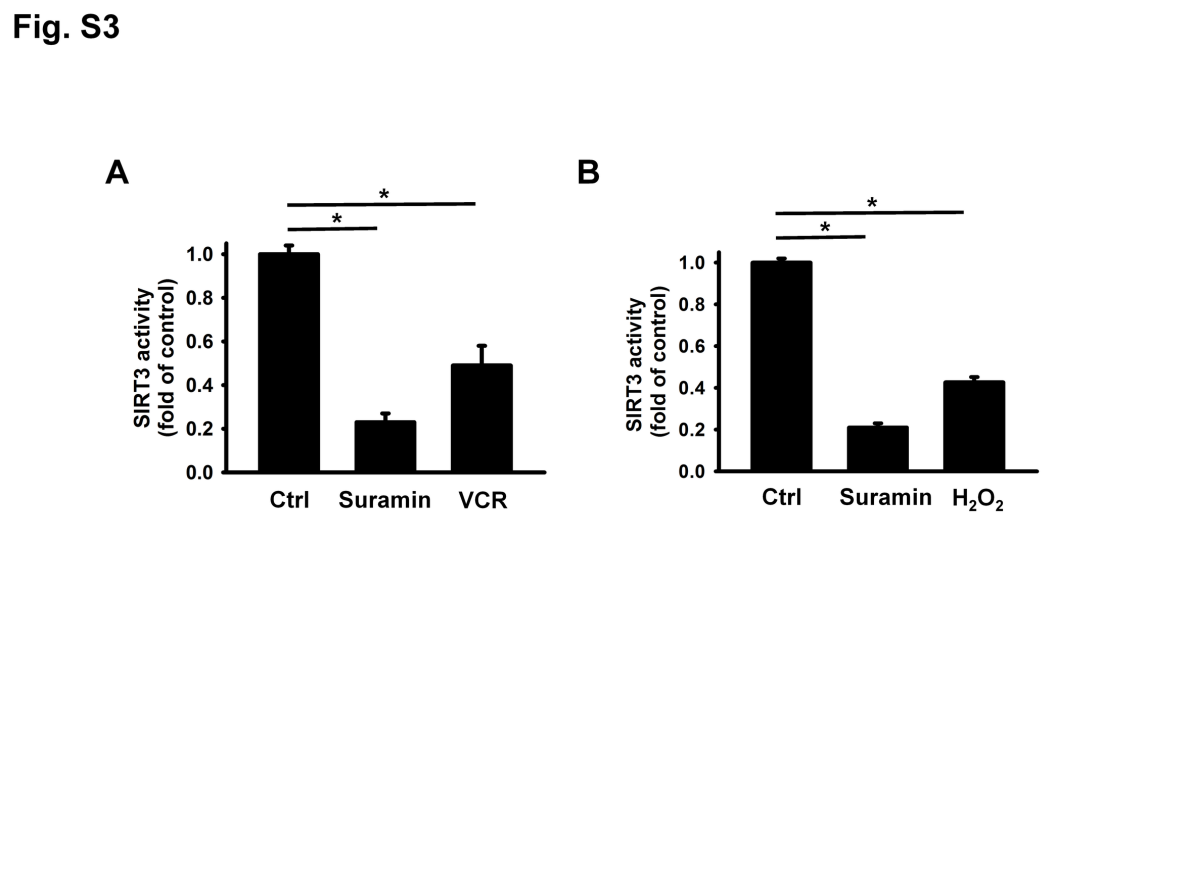


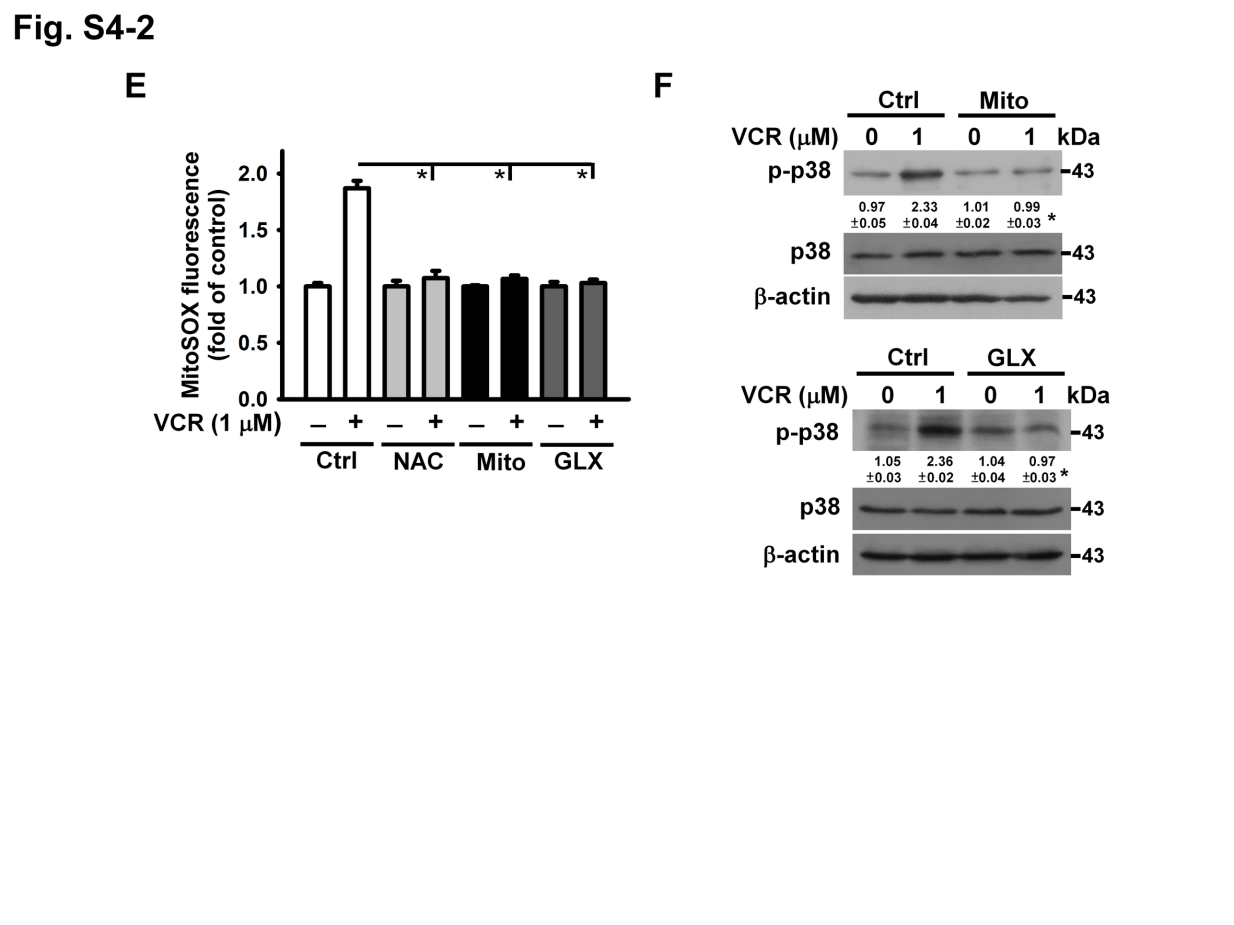

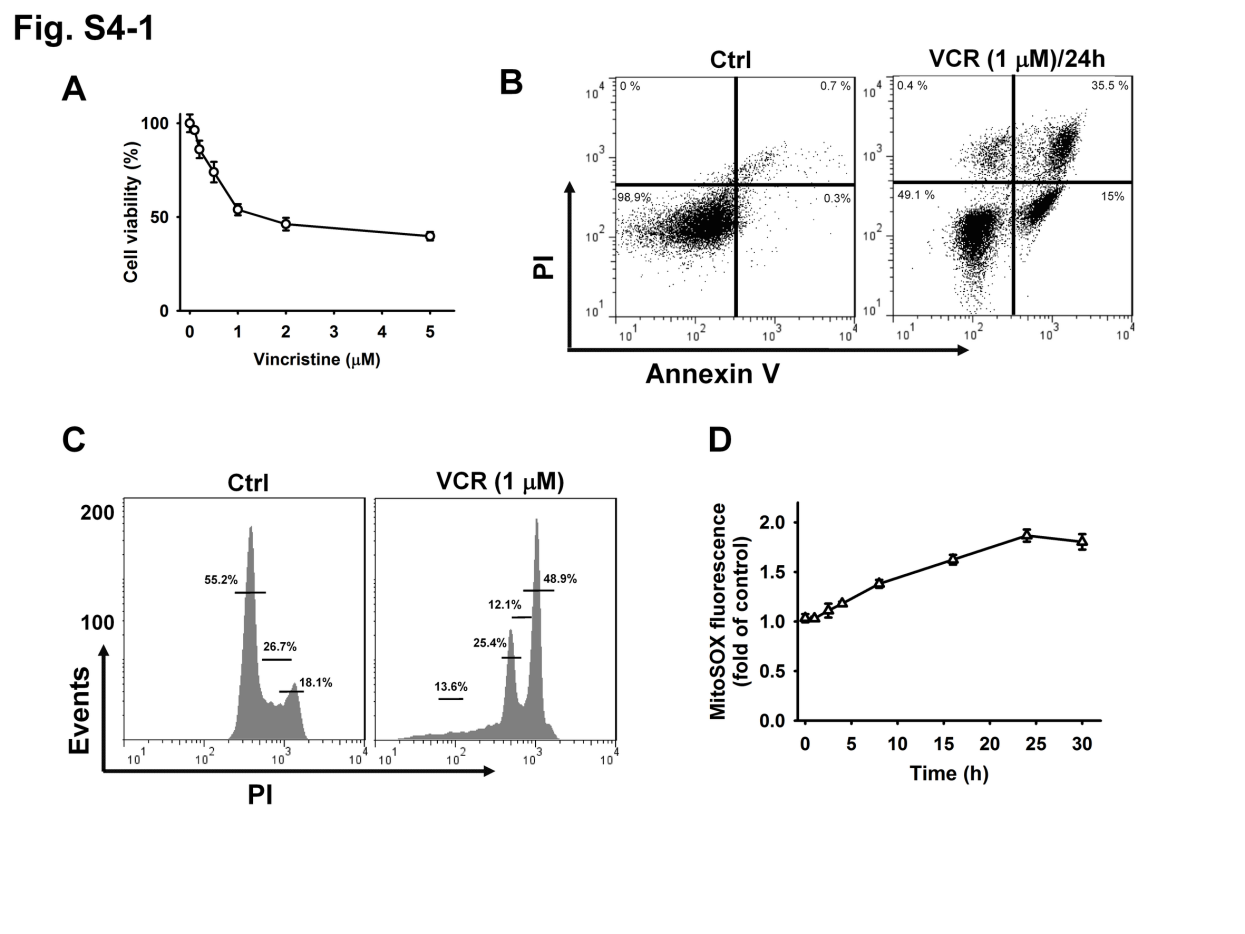

Supplement: Supplementary file 1 [file JCMM-24-2552-s001.docx]
